# Diagnosing the Dermatologic Blues: Systematic Review of the Rare Conundrum, Psychogenic Purpura

**DOI:** 10.2196/48153

**Published:** 2023-09-13

**Authors:** Praneet K Gill, Amy Zeglinski-Spinney

**Affiliations:** 1 Faculty of Medicine University of Ottawa Ottawa, ON Canada

**Keywords:** autoerythrocyte sensitization syndrome, dermatology, edema, female, gardner-diamond syndrome, painful purpura, psychocutaneous disorder, psychodermatology, psychogenic purpura, psychological stress, psychological stress, skin lesions, somatization, stress, stress-induced ecchymoses

## Abstract

**Background:**

Psychogenic purpura is an idiopathic psychodermatologic condition of recurrent, painful purpura precipitated by psychological stress, predominantly affecting young females. Little consensus exists on the diagnostic guidelines for this rare condition, often resulting in costly, unnecessary, and stressful investigations as well as prolonged hospital admissions.

**Objective:**

With this first up-to-date systematic review of 134 cases of psychogenic purpura in over a decade, we aim to thoroughly investigate the diagnostic strategy and treatment regimens used in the last decade. With a sooner diagnosis, patient stress and nosocomial ecchymoses can be minimized, and treatment can be expedited.

**Methods:**

We conducted a literature review of 4 databases (PubMed, Ovid Embase, Ovid MEDLINE, and Web of Science) on October 5, 2022 that yielded 46 full-text articles, which were reviewed and extracted by 2 independent reviewers.

**Results:**

We analyzed a total of 134 cases, consisting largely of females (125/134, 93.3%) with purpura on the upper (103/134, 76.9%) or lower limbs (112/134, 83.6%). Apart from a paresthesia prodrome, patients commonly experienced headaches, malaise, and arthralgia or myalgia. Approximately 70% (95/134) of patients reported a physiological or psychological stressor or psychiatric diagnosis before the development of the purpura. Laboratory testing almost always revealed unremarkable results. The intradermal washed autoerythrocyte sensitization test was positive in 98% (42/43) of cases. Histopathology biopsy findings commonly revealed dermal erythrodiapedesis or hemorrhage (n=34) and perivascular inflammatory infiltrates (n=17). Approximately 42% (56/134) of patients received a novel psychiatric diagnosis, with depression being the most common (40/72, 56%). In both patients with and those without a novel psychiatric diagnosis, observation, counseling, treatment with antidepressants (ie, selective serotonin reuptake inhibitors), and psychotherapy (ie, cognitive behavioral therapy) prevailed in the resolution of the purpura.

**Conclusions:**

Due to the unclear etiology and infrequent presentation of this condition, it remains a diagnosis of exclusion based on clinical suspicion evaluating the presence of stressors or psychiatric comorbidities and exclusion of systemic conditions. Clinical confirmation can be sought through a positive autoerythrocyte sedimentation test, characteristic histopathology findings, and remission of purpura after psychiatric treatment.

## Introduction

Psychogenic purpura (PP), also known as Gardner-Diamond syndrome or autoerythrocyte sensitization syndrome, is an idiopathic psychodermatologic condition in which patients exhibit recurrent, spontaneous, and painful purpura, often preceded by psychological distress or psychiatric comorbidity. The first case of autosensitization to a patient’s own blood was described by Gardner and Diamond [1], though PP-like dermatological manifestations of psychological factors have been described earlier by Schindler [2], where hypnosis of patients resulted in skin hemorrhages, and Jacobi [3], where patients with psychiatric comorbidities demonstrated purpura. Although the exact pathophysiology is unknown, it is hypothesized to be caused by psychological stress, estrogen, autoimmunity [4], low serotonin [5], and even religious “Holy Stigmata” [6]. It predominantly affects the young female demographic for reasons that remain unknown. Most challengingly, the clinical picture occurs in the absence of any pathognomonic homeostatic imbalances, with completely unremarkable hematologic, vascular, and immunologic results in most cases [7]. Histological results are generally characteristic of dermal hemorrhage but nonspecific. As a result, it has thus far been called a diagnosis of exclusion based on the clinical picture.

The aim of our systematic review of 134 cases is to describe the clinical presentation and most up-to-date diagnostic strategy of PP and provide a brief overview of treatment options available. To this end, we hope for improved patient experience and outcomes by reducing the duration of hospital admissions, unnecessary testing, and multiple physician and department transfers.

## Methods

This systematic review was completed according to the PRISMA (Preferred Reporting Items for Systematic Reviews and Meta-Analyses) guidelines. The PubMed, Ovid Embase, Ovid MEDLINE, and Web of Science databases were searched on October 5, 2022, for all relevant articles with the search terms “psychogenic purpura,” “Gardner Diamond Syndrome,” or “autoerythrocyte sensitization.” Using our searches, we hoped to address the following research questions: how do patients with PP present and how is it diagnosed? What level of evidence exists for the diagnosis and management of this disorder?

Articles that were not published in English were excluded. Research articles published before May 2013 were excluded on the basis of keeping our study relevant to current research published within the last 10 years. This is to prevent outdated medical and psychiatric knowledge from distorting pertinent data; importantly, the aim was to exclude out-of-date psychiatric perceptions and diagnoses from before the current version of the Diagnostic and Statistical Manual of Mental Disorders, Fifth Edition (DSM-5) was published on May 18, 2013. Due to the rarity of PP in the clinic, case reports were included and comprised a majority of the papers included in our review. Conference abstracts, posters, review articles, opinion articles that did not contain case reports or sufficient case information, and nonoriginal research were excluded.

Research articles pertaining to the diagnosis and clinical picture of PP were included. Initially, our search of the 4 databases revealed 727 articles; once duplicates were removed, 340 articles remained to be screened. All of the searched articles were reviewed by 2 independent reviewers (AZS and PKG). The Covidence systematic review software was used for article screening and data collection [8].

Title and abstract screening were performed using the inclusion and exclusion criteria given in Textbox 1.

**Inclusion and exclusion criteria.**

**Inclusion criteria**

- English
- Full text available
- Published within the last 10 years since the Diagnostic and Statistical Manual of Mental Disorders, Fifth Edition (DSM-5) publication (from May 18, 2013, to October 5, 2022)
- Original research article (ie, case reports, case-control, or cohort study)
- Study related to diagnosis and clinical presentation of psychogenic purpura

**Exclusion criteria**

- Review papers, opinion articles, and letters to the editor without a case report
- Full text unretrievable
- Research articles unrelated to the diagnosis and clinical presentation of psychogenic purpura
- Article not in English
- Article older than May 2013

**Figure 1:**
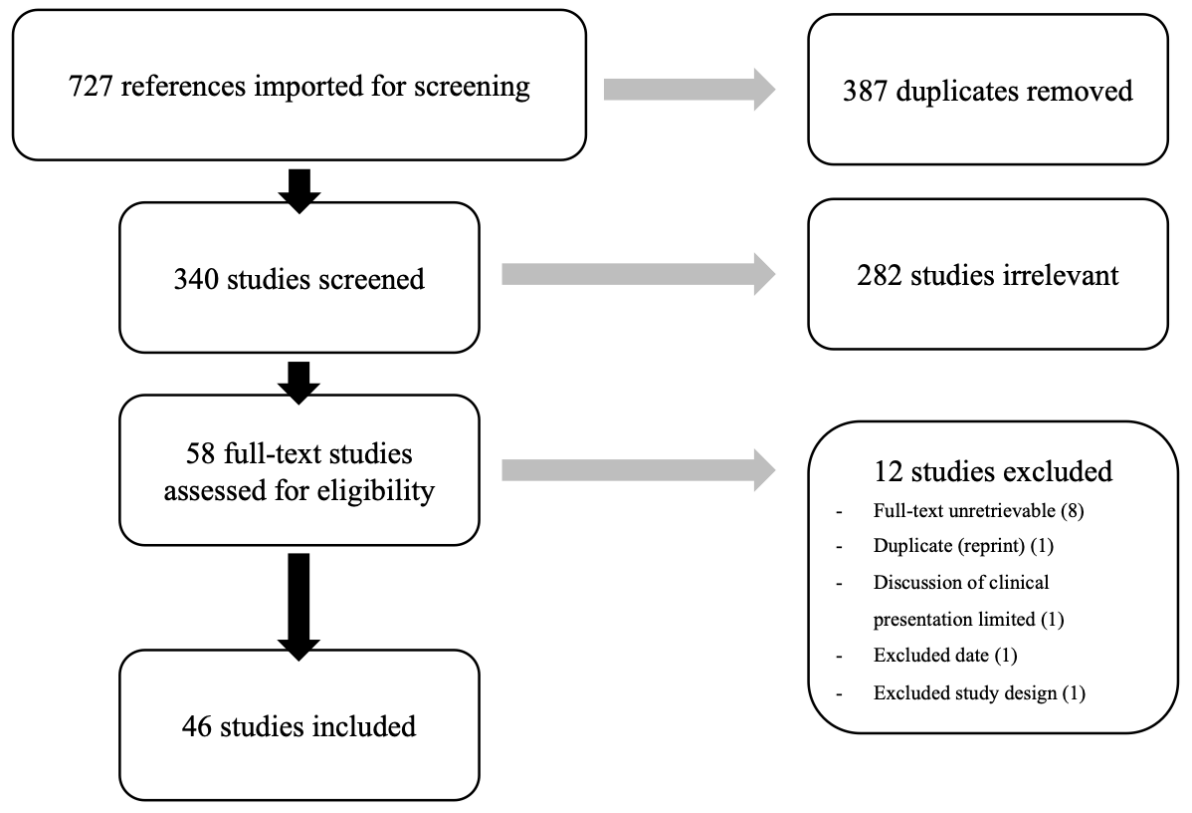
Title, abstract, and full-text screening flow chart.

After title and abstract screening, 282 studies were excluded. A total of 58 full texts were accessed and screened for eligibility. Subsequently, 12 full texts failed to meet the criteria and were excluded. In the end, 46 full texts remained and underwent review and data extraction (Figure 1).

Articles that passed full-text review were extracted for relevant data, as outlined below. Extracted data were exported into an Excel (version 16.59; Microsoft Corp) file. The data extracted from the papers are given in Textbox 2.

**Data extracted.**

**Demographic data**

- Type of study (case study, case-control, or randomized)
- Age of patients
- Sex of patients

**Clinical presentation data**

- Chief complaint
- Attending department (dermatology, psychiatry, family medicine, hematology, or internal medicine)
- Ruled out medication etiology
- Other etiologies ruled out (ie, factitious purpura or dermatitis artefacta, nonaccidental trauma, vasculitis, or panniculitis)
- Average size and number of purpura
- Pain with palpation of purpura
- Location of purpura
- Associated symptoms
- Past medical history
- Past social history (ie, low socioeconomic status and psychological or physiological stressor before the onset of purpura)
- Past family medical history (including of psychogenic purpura)

**Investigation data**

- Laboratory tests (ie, hemoglobin, erythrocyte sedimentation rate, platelet counts, prothrombin time and international normalized ratio, activated partial thromboplastin time, bleeding time, fibrinogen, antinuclear antibody, Coombs, cryoglobulin, anticardiolipin antibody, anti–double-stranded DNA, lupus anticoagulant, thrombocyte, and other abnormalities on peripheral blood smear)
- Histological findings from biopsy (ie, extravasation of erythrocytes in the dermis, perivascular infiltration of inflammatory cells, and dermal and subcutaneous hemorrhage)
- Autoerythrocyte sedimentation test

**Diagnosis and treatment data**

- New psychiatric diagnoses
- Treatment
- Remission status of purpura
- Other notes and important facts about the study

## Results

### Study and Patient Demographics

A total of 46 studies were included in our review [4,5,9-52], including 45 case studies and 1 retrospective study (Table S1 in Multimedia Appendix 1). A total of 134 cases were described in the case reports and retrospective study, of which 125 (95.4%) patients were female. The ages of the patients ranged from 8 years to 70 years. Among female cases where age was reported, 96% (49/51) of them were younger than 50 years.

### Patient Presentation

Patients commonly presented to dermatology or psychiatry, followed by hematology, rheumatology, pathology, pediatrics, internal medicine, or emergency medicine, with a history of purpura, often with pain upon palpation (112/134, 83.6%). The average duration of purpura was 16 months, ranging from 3 days to 6 years, and it was mostly found on the lower limbs (112/134, 83.6%) and the upper limbs (103/134, 76.9%; Table 1). Apart from a paresthesia prodrome, which was reported in 61% (70/114) of cases where symptoms were described, the symptoms accompanying the purpura were largely heterogeneous (Table 2). Other common symptoms included headache (31/114, 27%), malaise (30/114, 26%), arthralgia or myalgia (30/114, 26%), fever (17/114, 15%), and abdominal pain (15/114, 13%).

**Table 1:** Location of purpura in patients with psychogenic purpura (N=134).

Location of purpura	Patients, n (%)
Shoulders, arms, or hands	103 (76.9)
Legs or feet	112 (83.6)
Head or neck	41 (30.6)
Anterior trunk (abdomen or chest)	40 (29.9)
Posterior trunk (shoulders, dorsum, or buttocks)	32 (23.9)
Oropharyngeal	3 (2.2)
Unspecified	2 (1.5)

**Table 2:** Accompanying symptoms in patients with psychogenic purpura (N=114).

Accompanying symptoms	Patients, n (%)
Paresthesia (burning, tingling, or numbness)	70 (61.4)
Headache	31 (27.2)
Malaise	30 (26.3)
Arthralgia or myalgia	30 (26.3)
Fever	17 (14.9)
Abdominal pain	15 (13.2)
Fatigue or weakness	6 (5.3)
Epistaxis	4 (3.5)
Nausea	3 (2.6)
Vomiting	2 (1.8)
Pruritus	2 (1.8)
Hematuria	2 (1.8)
Hemoptysis	1 (0.9)
Hematemesis	1 (0.9)
Hematochezia	1 (0.9)
Bullae	1 (0.9)
Bleeding gums	1 (0.9)
Hemolacria	1 (0.9)
Urticaria	0 (0)
Other (“palpitations,” “dizziness,” “sore throat,” “edema,” “erythema,” “vasomotor changes like diaphoresis and hyperalgesia,” “aphthous stomatitis,” “miosis,” “conjunctival injection,” “lacrimation,” “night sweats,” “diarrhea,” “dysphagia,” “odynophagia,” “dyspnea,” “eye pain,” “loss of appetite,” “sleeping problems,” and “prodrome of bursting pain followed by swelling”)	10 (8.8)

Of 134 cases, just 1 case reported a family history of PP [5], and 2 cases reported low socioeconomic status [27,42]. Approximately 70% (95/134) of patients reported a physiological or psychiatric stressor before the development of the purpura. More specifically, 65.6% (88/134) of cases reported a psychological or physiological stressor before the onset of purpura (Textbox 3), and 27% (36/134) of cases had a previous psychiatric diagnosis.

**Types of stressors reported among 81 patients with psychogenic purpura.**

**General stress and anxiety**

**Home-related**

- Marital conflict or divorce
- Poor interpersonal relationships
- Parental divorce
- Stress related to the family member’s comorbidities

**Loss or bereavement**

- Child leaving for army
- Death of loved one
- Failed financial venture
- Living apart from spouse
- Relationship breakup
- Reduced interest in family members

**Maladaptive coping mechanisms**

- Binge drinking
- Self-harm

**Medical-related**

- Infection
- Hospitalization
- Hemodialysis
- Previous procedure or recovery
- Psychiatric comorbidity
- Surgery

**School-related**

- Academic decline
- Bullying
- Emotional distress
- Examinations—pressure of performance
- Learning delay
- Low self-esteem
- Performance anxiety

**Trauma**

- Abuse
- Natural disaster
- Sexual abuse

### Investigations and Diagnosis

On history, potential differential diagnoses (Textbox 4) were ruled out based on past medical and psychiatric history (Table S2 in Multimedia Appendix 1), and psychiatric and physiological stressors were investigated (Textbox 3), followed by laboratory investigations. Laboratory investigations, consisting of a combination of hemoglobin, erythrocyte sedimentation rate, platelet count, prothrombin time and international normalized ratio, partial thromboplastin time, bleeding time, fibrinogen, antinuclear antigen, Coombs test, cryoglobulin, anticardiolipin, anti–double-stranded DNA, and lupus anticoagulant, were negative in nearly all cases (84.4%-91.8%). Notable positives are listed in Supplemental Table S3 in Multimedia Appendix 1.

**List of alternative etiologies ruled out before the diagnosis with psychogenic purpura. Adapted from previous reviews [7,53].**

**Medication-induced**

- Antipsychotic medications
- Aspirin
- Nonsteroid anti-inflammatory drugs
- Recreational drug intake or abuse

**Vasculopathy**

- Deep vein thrombosis
- Cutaneous vasculitis (eg, nodular vasculitis, Henoch-Schonlein purpura, progressive pigmented purpura, cryoglobulinemia, angiitis, polyarteritis nodosa, superficial thrombophlebitis, capillaritis, such as disseminated pruriginous angiodermatitis)
- Panniculitis (eg, Pfeifer–Weber–Christian disease)

**Coagulopathy**

- Disseminated intravascular coagulation
- Idiopathic thrombocytopenic purpura
- Factor XIII deficiency
- Von Willebrand disease

**Connective tissue disorders** (eg, Ehlers-Danlos syndrome)

**Autoimmune conditions** (eg, systemic lupus erythematosus)

**Inflammatory dermatosis** (eg, stasis dermatitis)

**Other dermatological abnormalities with wood's lamp test** (eg, porphyria and ringworm)

**Hematological malignancy** (eg, cutaneous T-cell lymphoma)

**Other systemic illnesses** (eg, malignancy)

**Infectious process** (eg, atypical bacteria, tuberculosis, or deep mycoses)

**Trauma**

**Foreign body**

**Psychocutaneous conditions** (eg, factitious purpura or dermatitis artefacta)

Additional tests commonly completed include the autoerythrocyte sedimentation test and biopsy results. The autoerythrocyte sensitization test involves intradermally injecting autologous washed erythrocytes into the patient. A positive result is marked by the development of lesions after 24 hours alongside an absence of lesions in a negative control. The autoerythrocyte sensitization test is controversial due to its suspected low sensitivity and unknown specificity; nonetheless, it remains a popular diagnostic tool in the literature. In our cohort, 43 cases reported the use of the autoerythrocyte sensitization test to corroborate the diagnosis of PP, of which 42 had a positive result. These data suggest that the autoerythrocyte sensitization test equates to a sensitivity of 98% (Textbox S1 in Multimedia Appendix 1).

Histological findings from a biopsy of lesions demonstrated extravasation of erythrocytes in the dermis or dermal and subcutaneous hemorrhage (n=34). Next, perivascular infiltration of inflammatory cells was a frequent finding (n=17). Uncommon yet notable findings include 3 cases that reported hemosiderin pigment deposition in macrophages or dermis, 2 cases with fibrinoid deposition, and 2 cases with panniculitis. Other less common case-specific findings are summarized in Table S4 in Multimedia Appendix 1.

### Treatment

**Figure 2:**
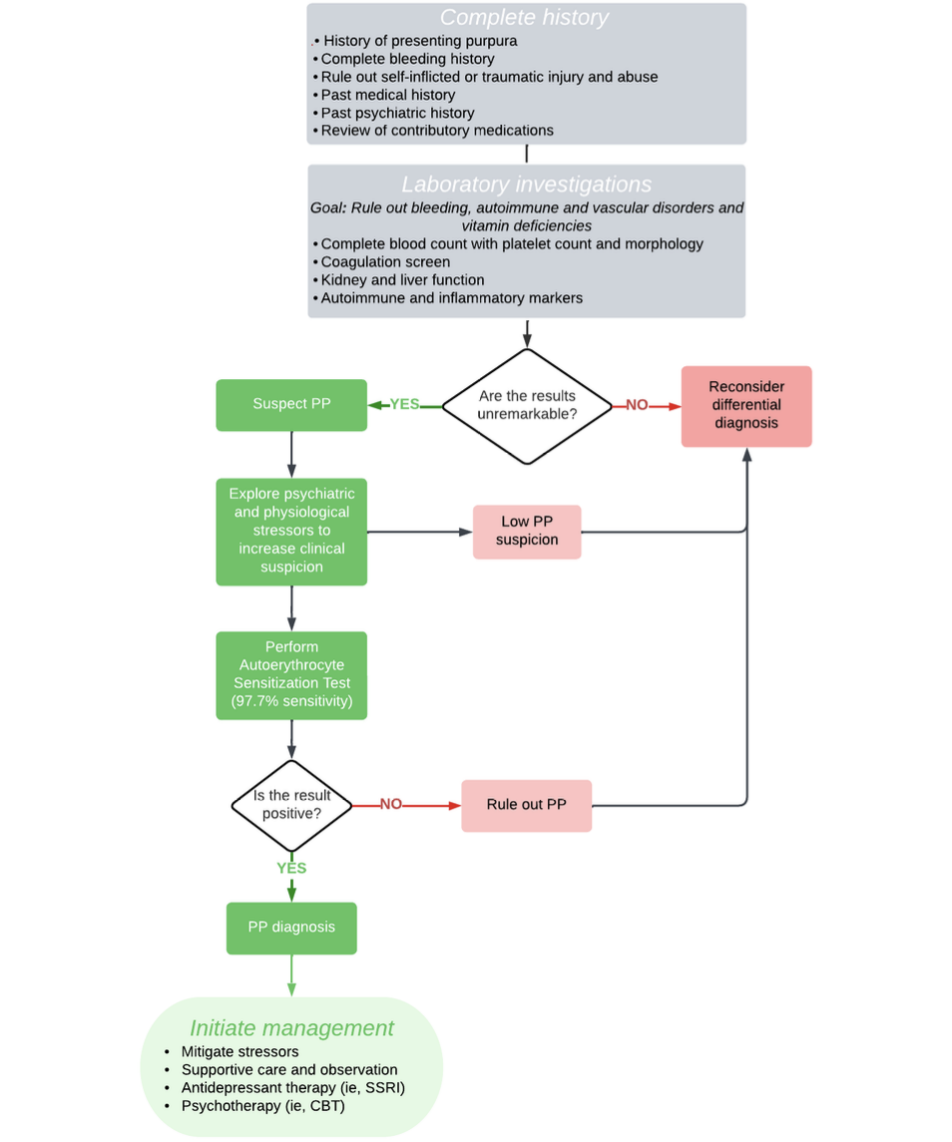
Proposed diagnostic algorithm for psychogenic purpura (PP). CBT: cognitive behavioral therapy; SSRI: selective serotonin reuptake inhibitor.

After diagnosis with PP, prompt treatment is crucial to address not only the etiology of the purpura but also the baseline psychological status of the patient. Some patients (56/134, 41.8%) received a novel psychiatric diagnosis in addition to PP (Figure 2). Depressive disorder was the most typical novel codiagnosis (40/72, 56%), followed by personality disorder (18/72, 25%), and anxiety disorder (9/72, 14%). The most common treatment administered to patients was observation, counseling, and support (50/127, 39%), followed by the commencement of antidepressant therapy (35/127, 27%), most commonly selective serotonin reuptake inhibitors (SSRIs; 23/127, 18%). Psychotherapy was frequently prescribed (25/127, 20%), followed by antihistamines (18/127, 14%), anti-inflammatory and immunosuppressant medications (16/127, 13%), and benzodiazepines (13/127, 10%). Historically, many other treatments have been used, including topical treatments and vitamins; these are summarized in Table S5 in Multimedia Appendix 1. Treatment practices have changed over time, with a greater emphasis on addressing psychological factors at present, likely due to the concomitant psychiatric comorbidities in patients with PP (Table 3). Before 2000, anti-inflammatory and immunosuppressant medications, such as nonsteroidal anti-inflammatory drugs, steroids, and hydroxychloroquine, were prescribed more often [18].

**Table 3:** Breakdown of novel comorbid Diagnostic and Statistical Manual of Mental Disorders, Fifth Edition (DSM-5) psychiatric diagnoses given to patients with psychogenic purpura. Note that some of these disorders overlap in the same patient and the total number of diagnoses exceed the total number of patients diagnosed with a psychiatric condition.

Psychiatric disorders	Patients (n=72), n (%)
Depressive disorder (ie, MDD^a^, other specified depressive disorder with anxious distress)	40 (56)
Personality disorder	18 (25)
Anxiety disorder (ie, generalized anxiety disorder, panic disorder panic disorder, or illness anxiety disorder)	9 (14)
Obsessive-compulsive disorder	1 (1)
Somatic symptom disorder	1 (1)
Conversion disorder	1 (1)
Trauma and stressor disorder (ie, adjustment disorder)	1 (1)
Unspecified mental disorder	1 (1)

^a^MDD: major depressive disorder.

Nearly all cases that were addressed with an effective treatment and had sufficient follow-up had initial remission of symptoms, with 74% of patients (n=52) having complete remission and 26% (n=18) having a relapse among cases with sufficient follow-up. Notably, of the 4 patients who did have a recurrence of PP and for which their treatment regimen was recorded, all refused or did not receive any psychotherapy (1 received oxygen inhalation and verapamil for pain [29], 1 received therapeutic plasma exchange [36], and 2 others received only SSRIs and benzodiazepines [47]). Incidentally, 1 patient completely refused all treatments and still experienced remission. Similarly, 63% of patients did not receive psychotherapy or counseling and still had full remission (n=30), while 38% of patients who had no therapy did have a relapse of PP (n=18). Interestingly, only 8% of patients (n=3) who received ≥1 form of psychiatric treatment (medication or therapy) had a relapse; in other words, 92% of patients (n=34) who received ≥1 form of psychiatric treatment (medication or therapy) had PP remission. Other comorbidities must also be adequately addressed as they come up; for example, 1 patient developed compartment syndrome that eventually required a fasciotomy [21].

## Discussion

Our study specifically focuses on the diagnosis and management of 134 cases published in the past decade, shining a fresh light on current practices. Similar to previous findings, we found PP presented predominantly in female patients younger than 50 years, accompanied by a prodrome of pain, burning, and paraesthesias, followed by rubor discoloration progressing into larger and darker ecchymotic lesions over the next 24-48 hours on the trunk, upper, or lower extremities [7,53,54]. While many patients presented with solely ecchymotic lesions, others presented with a plethora of other symptoms, such as headache, arthralgias, myalgia, fever, and abdominal pain [7]. Some patients had rarer prodromes, such as rapid progression of purpura on the anterior neck and oropharynx accompanied by dysphagia, odynophagia, and dyspnea [44], and another case with oral bleeding, epistaxis, and hematemesis recurring every 5-6 days [31]. However, none of the cases in our review reported internal organ bleeding, uterine hemorrhage, or renal hemorrhage despite reports of these in cases before May 2012 [54]. This study is the first up-to-date comprehensive review of PP in over a decade and one of the few to focus on the clinical presentation and diagnosis of this rare disorder. The past main reviews focused on treatment options [7] or were written before the DSM-5 was published [53].

Laboratory values are grossly normal in patients with PP, ruling out alternative etiologies [7]. Biopsy, when completed, commonly shows extravasation of erythrocytes in the dermis, perivascular infiltration of inflammatory cells, and dermal and subcutaneous hemorrhage [7], though it is not required for diagnosis [53]. The most common test for PP, sometimes referred to as the “gold standard,” is the washed autoerythrocyte sensitization test. In the cases included in our study, 98% (42/43) of cases tested had a positive result, compared with 85.7% (24/28) of cases tested in a previous review [7], demonstrating a relatively high test sensitivity. While PP can be diagnosed clinically in most cases, this test can be used in instances of clinical uncertainty. Our results suggest that the autoerythrocyte sensitization test has a high sensitivity of 98% for PP. With this new finding, we propose the following diagnostic algorithm for PP (Figure 2). Clinical judgment should be exercised. Due to the test’s historically postulated low sensitivity in the literature, a negative test may not always rule out PP if the clinical picture and investigations both support the diagnosis.

A large proportion of patients have a psychological stressor before the onset of the purpura (Textbox 3), and many have a concomitant psychiatric disorder, most commonly depression [7,53] (Table 3), necessitating psychiatric assessment [55]. In a previous review, 93% of cases had either a stressor or psychiatric comorbidity, relative to 70% (95/134) in our study. After diagnosis, patients are successfully treated with supportive counseling, psychotherapy, or antidepressants (ie, SSRIs) tailored to address the underlying psychiatric comorbidities and stressors [53,54]. Purpuric lesions tend to resolve alongside a psychiatric response to therapy or mitigation of stressors [7]. Additional therapies, such as antihistamines, steroids, and other topical agents, may also be prescribed based on symptoms [53]. Although PP follows a chronic disease course with relapses after trauma or stress, many patients achieve remission with favorable prognosis [53,54]. Mental illness can have a significant psychogenic impact on dermatology, as seen in the strong correlation between anxiety and mood disorders with acne, eczema, psoriasis, psychogenic pruritus, and psychogenic excoriation [56]. Dermatologic conditions and their aftermath, including scars, can in turn affect and exacerbate self-esteem, body image, and psychological distress [57,58]. A study reported suicidal ideation in 67.6% of patients with psoriasis and 68% of patients with atopic dermatitis [59]. This can create a vicious cycle, which is very clearly evident in the case of PP.

The appearance of purpura and subsequent social stigma can trigger psychological distress, worsening physical symptoms. The distress can become economically taxing, with time taken away from school or work and frequent health care–seeking behaviors. Extended time in hospital until diagnosis can exacerbate illness anxiety and precipitate additional nosocomial purpura. Prompt intervention and reassurance can stop this chain of events. In patients with PP overly distraught with their purpura, somatic symptom disorder or illness anxiety disorder may be additionally considered on the differential [60]. Comparably, due to symptomology overlap, PP could be characterized as a variant of the DSM-5’s conversion disorder. Care providers should recognize the dynamic relationship between mental health and dermatology and collaborate with mental health professionals. Each person’s experience with PP is unique; some individuals may have better coping mechanisms and support systems that help mitigate the challenges of living with this condition, while others may need increased support and resources.

Our data are limited by the 45 case reports and single retrospective study included. Case studies are heterogeneous, limited in statistical inference strength, and inconsistent. For example, not all papers tested for autoerythrocyte sensitization, limiting our ability to conduct a meta-analysis. Furthermore, our data may exclude clinical presentations that were misdiagnosed, unreported, undiagnosed due to a mild course, or experienced by individuals with health inequities lacking access to care. In addition, due to the limited number of cases in the literature and the criterion of excluding outdated papers from before May 2013, external validity may be attenuated. For this reason, our high sensitivity calculation of the autoerythrocyte sensitization test does not serve as enough evidence to recommend screening all patients with purpura but rather should enlighten physicians that the test may have an important role in the differential diagnosis toolkit.

Our study also has various strengths, notably its comprehensiveness in including both case reports and a retrospective study, as well as dissemination by 2 independent reviewers from 4 different databases, reducing selection bias and increasing reliability, reproducibility, and validity.

The pathophysiology of PP remains to be fully established; however, there are numerous theories implicating immune processes, the kallikrein-kinin system, and vascular properties. Female predominance of PP suggests involvement of estrogen and an autoimmune component [4], which is supported by the successful treatment of 1 case with plasmapheresis [61]; however, a case in our study treated by plasmapheresis did not have remission of purpura [36]. Incubation of erythrocytes from healthy individuals in the plasma of patients with PP containing immunoglobulin E to cardiolipin and phosphatidylserine, the phosphoglyceride of erythrocyte membranes, results in greater than 50% erythrocyte phosphatidylserine to be redistributed on the outer surface of the cell membrane [62]. This builds on the original theory that PP results from autosensitization to phosphatidylserine [1,63]. Abnormal tonus regulation of venous capillaries by the kallikrein-kinin system may also be involved, as well as extravasation of erythrocytes with sensitizing antibodies, defective synthesis of fibrin in the endothelium, and structures in the capillary wall [64]. Capillaritis, characterized by an inflammatory infiltrate of lymphocytes, just like some patients with PP, involves vascular fragility in the pathogenesis. This vascular fragility may occur in PP, whereby lymphocytes may interact with the vascular endothelium to affect permeability [65]. The role of low serotonin in the disease process has been speculated as SSRIs are effective in remitting purpura and preventing disease relapse. Serotonin, which is low in mood and anxiety disorders, is stored in platelets and plays a vital role in hemostatic aggregation and coagulation pathways, as well as reflexive vasoconstriction. Hemorrhage risk increases with peripheral serotonin depletion [66], possibly contributing to purpura. Many hypotheses exist on the pathophysiology of PP, but the evidence is lacking. Pertinent future research should investigate platelet or serum serotonin levels, autoimmunity, and vascular elements in patients with PP to further our understanding behind the disease process.

Ultimately, the diagnosis of PP is based on a thorough clinical history and physical examination, along with normal laboratory investigations. Diagnostic certainty is aided by the resolution or regression of purpura following the commencement of psychiatric therapy. A positive autoerythrocyte sensitization test and histological findings further support the diagnosis in times of clinical uncertainty. Increasing understanding and developing an evidence-based approach to managing this psychodermatologic condition can improve patient outcomes through earlier and appropriate psychiatric treatment interventions. A staggering number of cases demonstrate the repercussions of unfocused, prolonged differential investigations delaying diagnosis, leading to worsened purpura, physical symptoms, and psychological stress. Thus, physicians must remember to factor in a patient’s mental health when treating skin conditions and recognize dermatological manifestations of psychological status. Patients can receive more effective and comprehensive care by addressing both the physical and psychological aspects of skin conditions.

## Appendix

Multimedia Appendix 1 Supplemental material.

Multimedia Appendix 2 PRISMA checklist.

## Abbreviations

DSM-5: Diagnostic and Statistical Manual of Mental Disorders, Fifth Edition
PP: psychogenic purpura
PRISMA: Preferred Reporting Items for Systematic Reviews and Meta-Analyses
SSRI: selective serotonin reuptake inhibitor
