# Supplementary material for: Diagnosing the Dermatologic Blues: Systematic Review of the Rare Conundrum, Psychogenic Purpura
Source: JMIR Dermatol. 2023 Sep 13;6:e48153. doi: 10.2196/48153 (PMC10534291; doi:10.2196/48153)
Supplement: Multimedia Appendix 1 [file derma_v6i1e48153_app1.docx]

Multimedia Appendix 1

Textbox S1:

Calculations for sensitivity of the autoerythrocyte sensitization test

Sensitivity calculation = True Positive/(True Positive + False Negative) × 100%

= 42/(42 + 1) × 100%

= 97.67%

Table S1: Demographic data of included studies.

| Author & Year | Title | Country | Type of Study | Patient age | Number of patients | |
| --- | --- | --- | --- | --- | --- | --- |
|  |  |  |  |  | Female | Male |
| Amichai et al. 2013 [10] | Psychogenic Purpura (Gardner-Diamond syndrome) - Recurrent spontaneous painful ecchymosis and systemic symptoms | Israel | Case study | 16 |  | 1 |
| Thomas et al. 2014 [11] | An unusual case of painful purpura-Gardner-Diamond Syndrome | United Kingdom | Case study | 13 | 1 |  |
| Estrada et al. 2016 [12] | Gardner-Diamond Syndrome as a differential diagnosis of lupus | Columbia | Case study | 23 | 1 |  |
| Amouri et al. 2020 [13] | Gardner-Diamond Syndrome: clinical and biological approach (2 cases) | Tunisia | Case study | 29, 20 | 2 |  |
| Marques et al. 2022 [9] | Psychogenic Purpura - remembering a rare image | Portugal | Case study | 26 | 1 |  |
| Singh et al. 2021 [14] | Diamond Gardner Syndrome in a male: a case report | India | Case study | 32 |  | 1 |
| Krajewski et al. 2022 [15] | Recurrent spontaneous petechiae and ecchymoses in a 14-year-old girl | Poland | Case study | 14 | 1 |  |
| Kocoglu et al. 2022 [16] | A rare cause of severe ecchymosis: 'Diamond-Gardner Syndrome' | Turkey | Case study | 45 |  | 1 |
| Gözdaşoğlu 2013 [17] | Autoerythrocyte Sensitization Syndrome treated with Kallikrein inhibitor | Turkey | Case study | 20 | 1 |  |
| Sridharan et al. 2019 [18] | The Mayo Clinic experience with Psychogenic Purpura (Gardner-Diamond Syndrome). | United States | Retrospective study | 15-69 | 74 | 2 |
| Okeson et al. 2022 [19] | Adolescent with leg rash | United States | Case study | 17 | 1 |  |
| Akar et al. 2019 [20] | Diamond-Gardner Syndrome: Autoerythrocyte Sensitization Syndrome | Turkey | Case study | 16 | 1 |  |
| Sen et al. 2015 [65] | Gardner Diamond Syndrome: a case report and brief review | United Kingdom | Case study | 42 | 1 |  |
| Iskandarli et al. 2016 [66] | Gardner-Diamond Syndrome in a pediatric patient | Turkey | Case study | 8 | 1 |  |
| Sen et al. 2015 [21] | Bruising, bleeding and recurrent compartment syndrome: a case of Gardner-Diamond Syndrome (Autoerythrocyte Sensitization Syndrome)? | United Kingdom | Case study | 12 | 1 |  |
| Chakraborty et al. 2016 [22] | A case vignette Of Auto Erythrocyte Sensitization (Gardner Diamond) Syndrome presenting in the outpatient department of a rural hospital | India | Case study | 28, 44 | 2 |  |
| Arora et al. 2013 [23] | Bullous Auto Erythrocyte Sensitization Syndrome in alcohol dependence. | India | Case study | 25 |  | 1 |
| Jafferany 2013 [24] | Auto-Erythrocyte Sensitization syndrome (Gardner-Diamond syndrome) in a 15-year-old adolescent girl. | United States | Case study | 15 | 1 |  |
| Sawant et al. 2012 [25] | Antidepressant-induced remission of Gardner Diamond Syndrome. | India | Case study | 25 | 1 |  |
| Nasu et al. 2013 [26] | A child case of Gardner-Diamond Syndrome triggered by the Tohoku-Pacific ocean earthquake. | Japan | Case study | 11 | 1 |  |
| Sarkar et al. 2013 [27] | Psychogenic Purpura. | India | Case study | 45 | 1 |  |
| Oh et al. 2013 [28] | Autoerythrocyte Sensitization Syndrome presenting with general neurodermatitis. | South Korea | Case study | 70 | 1 |  |
| Silva et al. 2014 [29] | Bloody tears, Gardner-Diamond Syndrome, and trigemino-autonomic headache. | Brazil | Case study | 38 | 1 |  |
| Tainwala et al. 2013 [30] | Perplexing purpura in two females: rare case of Autoerythrocyte Sensitization Syndrome. | India | Case study | 19, 30 | 2 |  |
| Karakaş et al. 2014 [5] | A disease difficult to diagnose: Gardner-Diamond Syndrome accompanied by platelet dysfunction. | Turkey | Case study | 13 | 1 |  |
| Qamar et al. 2015 [31] | Per oral bleeding: rare presentation of Gardner-Diamond Syndrome. | Pakistan | Case study | 10 |  | 1 |
| Jafferany et al. 2015 [32] | Psychogenic Purpura (Gardner-Diamond Syndrome). | United States | Case study | 15 | 1 |  |
| Park et al. 2016 [33] | Gardner-Diamond Syndrome. | United States | Case study | 27 | 1 |  |
| Woo et al. 2016 [34] | Recurrent purpuric patches on the limbs of an 18-year-old-female: Gardner-Diamond Syndrome. | South Korea | Case study | 18 | 1 |  |
| Bizzi et al. 2016 [35] | A long-term psychological observation in an adolescent affected with Gardner Diamond Syndrome. | Italy | Case study | 16 | 1 |  |
| Millward et al. 2017 [36] | Management of Gardner-Diamond syndrome with therapeutic plasma exchange. | United States | Case study | 30 | 1 |  |
| Çelik-Göksoy et al. 2017 [37] | Psychogenic Purpura successfully treated with antidepressant therapy. | Turkey | Case study | 15 | 1 |  |
| Vivekanandh et al. 2017 [38] | Gardner Diamond Syndrome: a psychogenic purpura. | India | Case study | 35 | 1 |  |
| Thokchom et al. 2018 [39] | Clinical spectrum of Autoerythrocyte Sensitization Syndrome: a series of five cases. | India | Case study | 29, 25, 22, 38, 45 | 5 |  |
| Dabas et al. 2018 [40] | Inexplicable purpura in a female: Gardner-Diamond Syndrome. | India | Case study | 39 | 1 |  |
| Kara et al. 2019 [41] | Gardner-Diamond Syndrome in an adolescent with suicidal ideation: a case report. | Turkey | Case study | 14 | 1 |  |
| Ferizi & Gercari 2019 [42] | Psychogenic purpura. | Kosovo | Case study | 20 | 1 |  |
| Dick et al. 2019 [43] | Gardner-Diamond Syndrome: a psychodermatological condition in the setting of immunodeficiency. | United States | Case study | 32 | 1 |  |
| Allar et al. 2020 [44] | Oropharyngeal Psychogenic Purpura. | United States | Case study | 57 | 1 |  |
| Bellot et al. 2020 [45] | Oral management in a patient with Gardner-Diamond Syndrome: A case report. | France | Case study | 35 |  | 1 |
| Akoglu et al. 2021 [46] | Psychogenic Purpura (Gardner-Diamond Syndrome) in a hemodialysis patient. | Turkey | Case study | 39 | 1 |  |
| Temiz et al. 2021 [4] | Is Gardner-Diamond Syndrome related to autoimmunity? | Turkey | Case study | 26 | 1 |  |
| Priyam et al. 2021 [47] | Psychiatric profile and response to combined pharmacologic and psychotherapeutic treatment in Psychogenic Purpura. | India | Case study | 14, 24, 70, 35, 29, 22, 18 | 7 |  |
| Balcioglu et al. 2021 [48] | A severe form of Gardner-Diamond Syndrome in a patient with schizophrenia: a rare comorbidity with an exceptional pathophysiology. | Turkey | Case study | 45 |  | 1 |
| Harada & Hagiwara 2022 [49] | Gardner-Diamond Syndrome complicated with chronic urticaria successfully treated with antihistamine. | Japan | Case study | 19 | 1 |  |
| Khadke et al. 2022 [50] | Gardner-Diamond Syndrome in an adolescent girl. | India | Case study | 18 | 1 |  |

Table S2: Past medical and psychiatric history found among cases.

| Past medical history | Ref | Past psychiatric history | Reference |
| --- | --- | --- | --- |
| Chronic anemia | [45] | Anxiety disorder | [14,36,39,45,46,65] |
| Chronic neurodermatitis | [28] | Bipolar affective disorder | [16] |
| Common variable immunodeficiency |  | Depression | [9,15,22,27,37,39,43] |
| Compartment syndrome | [11,21] | Eating disorder | [35] |
| Fibromyalgia | [43] | Hyperactivity and hysterical behavior | [66] |
| Inflammatory bowel syndrome | [4] | Obsessive compulsive disorder | [33] |
| Juvenile idiopathic arthritis & amplified musculoskeletal pain syndrome | [19] | Personality disorder | [15] |
| Hashimoto’s disease | [4] | PTSD | [26,37] |
| Hypertension | [46] | Substance use disorder | [23] |
| Menstrual disturbance (i.e., heavy menstruation, amenorrhea) | [65] | Schizophrenia | [16,48] |
| Migraines | [36] |  |  |
| Neurological diagnoses | [18] |  |  |
| Psychogenic purpura | [25,44] |  |  |
| Pulmonary embolism | [45] |  |  |
| Regional pain syndrome | [21] |  |  |
| Severe asthma | [45] |  |  |
| Systemic lupus erythematosus | [12] |  |  |

Table S3: Notable positive laboratory values.

| Number of Cases | Laboratory Measurement | Reference |
| --- | --- | --- |
| 4 | Anemia | [33,36,39,45] |
| 1 | High erythrocyte sedimentation rate | [39] |
| 1 | Low aPTT | [18] |
| 1 | High bleeding time | [12] |
| 3 | Positive ANA | [4,21,50] |
| 1 | Positive anti-dsDNA | [4] |
| 1 | High VWF:Ag level | [18] |

Table S4: Histological findings from biopsies. Note that there is overlap in biopsy findings in individual patients.

| Number of Cases | Histological Finding | Reference |
| --- | --- | --- |
| 34 | Extravasation of erythrocytes in the dermis and/or dermal and/or subcutaneous hemorrhage | [4,10,12,13,15,16,18,23,26,30,33,34,37,43,46,48,66] |
| 17 | Perivascular infiltration of inflammatory cells | [4,10,13,18,21,23,28,30,33,34,46,66] |
| 3 | Hemosiderin pigment deposition in macrophages or dermis | [23,33,42] |
| 2 | Fibrinoid deposition around blood vessels | [4,30] |
| 2 | Panniculitis | [18] |
| 1 | Orthokeratotic epidermis with hyperpigmentation of basal layer. | [15] |
| 1 | Thinning of the epidermis, vascular proliferation, thick-walled vascular structure in the subcutaneous fatty tissue. | [16] |
| 1 | Endothelial accentuation. | [66] |
|  |  |  |
| 1 | Non-specific inflammatory reaction pattern | [34] |

Table S5: Treatment regimens reported in 127 patients with psychogenic purpura. Note that there is overlap in treatments in individual patients.

| **Treatment** | | **Number of cases** | **Reference** |
| --- | --- | --- | --- |
| Psychological observation, counseling, and support | | 50 | [10,17,18,50] |
|  | Consultation with teachers and psychologist | 1 | [10] |
|  | Subsequent psychological support after recovery | 1 | [17] |
|  | Regular counseling | 1 | [50] |
|  | Counseling to address psychological stressors & observation | 47 | [18] |
| Antidepressants | | 35 | [14,17,30,31,35,38] |
|  | SSRI (sertraline, fluoxetine, cetirizine, escitalopram, paroxetine) | 23 | [12,15,16,19,22,24,25,27,30,32,37,41,45–48,66] |
|  | Unspecified antidepressant | 5 | [5,18] |
|  | TCA (i.e., amitriptyline, nortriptyline) | 5 | [14,30,31,35,38] |
|  | Atypical antidepressant (i.e., mirtazapine) | 1 | [65] |
|  | SNRI (i.e., venlafaxine) | 1 | [22] |
| Psychotherapy (i.e., CBT) | | 25 | [5,14–16,23,35,39,41,44,47,48,65] |
| Antihistamine (i.e., cyproheptadine, bepotastine besilate) | | 18 | [16,18,28,42,49,50] |
| Oral anti-inflammatory/immunosuppressants (i.e., NSAIDS, steroids, hydroxychloroquine, rituximab) | | 16 | [4,18,28,50] |
| Benzodiazepines (i.e., lorazepam, clonazepam) | | 13 | [14,22,30,38,42,47] |
| Topical treatments | | 5 | [4,33,42,66] |
|  | Topical corticosteroid | 2 | [42,66] |
|  | Sarna lotion | 1 | [33] |
|  | Hepathrombin cream | 1 | [42] |
|  | Mucopolysaccharide polysulfate cream | 1 | [4] |
| Vitamin supplementation | | 4 | [12,16,23,42] |
|  | Unspecified | 1 | [23] |
|  | 2% Vitamin K | 1 | [12] |
|  | Vitamin C | 2 | [16,42] |
| Other | |  |  |
|  | Hormonal medications (i.e., cyclic estrogen and oral contraceptives) | 2 | [18] |
|  | Anticonvulsant (i.e., topiramate, pregabalin) | 2 | [23,65] |
|  | Dapsone | 2 | [18] |
|  | Abstinence from alcohol & naltrexone | 1 | [23] |
|  | Antipsychotic (i.e., risperidone) | 1 | [48] |
|  | Anxiolytics | 1 | [28] |
|  | DDAVP | 1 | [11] |
|  | Erythrocyte transfusion | 1 | [16] |
|  | Kallikrein inhibitor aprotinin | 1 | [17] |
|  | Relaxation exercises | 1 | [65] |
